# Determinants of acute respiratory infection among under-five children in rural Ethiopia

**DOI:** 10.1186/s12879-021-06864-4

**Published:** 2021-11-30

**Authors:** Amanuel Mengistu Merera

**Affiliations:** Department of Statistics, College of Natural and Computational Science, Mizan-Tepi University, Tepi, Ethiopia

**Keywords:** Acute respiratory infections, Under-five children, Logistic Regression, EDHS

## Abstract

**Introduction:**

In low- and middle-income nations, acute respiratory infection (ARI) is the primary cause of morbidity and mortality. According to some studies, Ethiopia has a higher prevalence of childhood acute respiratory infection, ranging from 16 to 33.5%. The goal of this study was to determine the risk factors for acute respiratory infection in children under the age of five in rural Ethiopia.

**Methods:**

A cross-sectional study involving 7911 children under the age of five from rural Ethiopia was carried out from January 18 to June 27, 2016. A two stage cluster sampling technique was used recruit study subjects and SPSS version 20 was used to extract and analyze data. A binary logistic regression model was used to identify factors associated with a childhood acute respiratory infection. The multivariable logistic regression analysis includes variables with a p-value less than 0.2 during the bivariate logistic regression analysis. Adjusted odds ratios were used as measures of effect with a 95% confidence interval (CI) and variables with a p-value less than 0.05 were considered as significantly associated with an acute respiratory infection.

**Results:**

The total ARI prevalence rate among 7911 under-five children from rural Ethiopia was 7.8%, according to the findings of the study. The highest prevalence of ARI was found in Oromia (12.8%), followed by Tigray (12.7%), with the lowest frequency found in Benishangul Gumuz (2.4%). A multivariable logistic regression model revealed that child from Poor household (AOR = 2.170, 95% CI: 1.631–2.887), mother’s no education (AOR = 2.050,95% CI: 1.017–4.133), mother’s Primary education (AOR = 2.387, 95% CI:1.176–4.845), child had not received vitamin A (AOR = 1.926, 95% CI:1.578–2.351), child had no diarrhea (AOR = 0.257, 95% CI: 0.210–0.314), mothers not working (AOR = 0.773, 95% CI:0.630–0.948), not stunted (AOR = 0.663, 95% CI: 0.552–0.796), and not improved water source (AOR = 1.715, 95% CI: 1.395–2.109). Similarly, among under-five children, the age of the child, the month of data collection, anemia status, and the province were all substantially linked to ARI.

**Conclusions:**

Childhood ARI morbidity is a serious health challenge in rural Ethiopia, according to this study, with demographic, socioeconomic, nutritional, health, and environmental factors all having a role. As a result, regional governments, healthcare staff, and concerned groups should place a priority on reducing ARI, and attempts to solve the issue should take these variables into account.

## Introduction

ARI is an infection that makes it difficult to breathe normally. Acute Upper Respiratory Infections and Acute Lower Respiratory Infections are the two forms of Acute Respiratory Infections based on the site of infection [1]. The airways from the nose to the voice cords within the larynx make up the upper tract.

The continuation of the airways from the trachea and bronchi to the bronchioles, as well as the alveoli, is part of the lower tract [2, 3]. In low- and middle-income nations, respiratory illness is the major cause of morbidity and mortality [4] across all age groups and sexes [5]. In 2016, lower respiratory infections were responsible for 652, 572 deaths in children under the age of five globally [6]. Acute lower respiratory tract infection in the form of pneumonia, in particular, is regarded as the leading cause of childhood death worldwide, accounting for 16 percent of all deaths in 2015 [7].

Acute respiratory tract infection is one of the most common childhood illnesses, and it almost invariably results in major health problems and death in children under the age of five [8]. ARIs were blamed for 73 percent of the estimated 10.4 million deaths of children under the age of five that occurred over the world [9]. In the WHO African Region, the under-five death rate is 74/1000 live births, over eight times higher than in the WHO European Region (9/1000 live births) [8]. According to the 2016 Ethiopian Demographic and Health Surveys, the under-five mortality rate in Ethiopia is 67/1000 live births [10]. Each year, four to five bouts of ARI affect children under the age of five. ARI is responsible for 30–50% of visits to health institutions and 20–40% of hospital admissions for children under the age of five [11].

Acute Lower respiratory infections (ALRI) were the leading cause of premature mortality across all ages in Ethiopia [12] and pneumonia, in particular, was the second most common cause of mortality in Northeast Ethiopia [13]. Numerous studies have found that socioeconomic and other characteristics, such as the child's age, household income, environmental conditions, parental education, maternal age, and other factors, are linked to ARI [14–18]. In addition, many cross-sectional studies conducted in different parts of the country reported an even higher prevalence of childhood ARI in Ethiopia which ranges from 16% up to 33.5% [19–23]. 

The majority of factors of ARI occur in rural areas and among children under one year of age (Mirji et al. 2014). Several studies showed that Children from rural setups were more prone to develop ARI [24, 25]. The probable explanation for the greater ARI symptoms proportion for rural children may be due to lack of access to medical treatment, low socio-economic standards in rural regions [26]. According to the Ethiopian demographic and health survey (EDHS), the prevalence of ARI among under-five children was 13% in 2005, and therefore the magnitude reduced to 7% in EDHS 2011, however, the prevalence remained 7% in EDHS 2016. So far, no study had been done on determinants of ARI in different rural communities of Ethiopia and contributing factors for the disease were not well explored. Therefore, ARI cannot be tackled without understanding its causes that is why the study is crucial to investigate the major socio-economic, demographic, health, environmental and nutritional related determinate of ARI among under-five children in rural Ethiopia.

## Methods and materials

### Study design and setting

A cross-sectional study was conducted from January 18 to June 27, 2016. The study was conducted in Ethiopia, located in the Horn of Africa between 3 and 15 degrees’ north latitude and 33 and 48 degrees’ east longitude. Ethiopia is bordered by Eritrea to the North, Djibouti, and Somalia to the East, Sudan and South Sudan to the West, and Kenya to the South. Ethiopia has eleven geographic or administrative regions; it includes nine regional states and two city administrations. The dataset in this study was obtained from the Demographic and Health Survey conducted in Ethiopia in 2016. The 2016 EDHS was the fourth survey conducted in Ethiopia as part of the worldwide Demographic and Health Surveys project. It was conducted by the Central Statistical Agency (CSA) at the request of the Federal Ministry of Health (FMoH).

### Population and sampling procedures

The 2016 EDHS sample was selected by considering two-stage cluster design and census enumeration areas (EAs) were the sampling units for the first stage. A typical two-level stratification involves first stratifying the population by region at the first level, next by urban–rural within each region. The sample included 645 EAs (202 in urban areas and 443 in rural areas). In the sampling procedure, households comprised the second stage of sampling. A complete listing of households was carried out in each of the 645 selected enumeration areas by equal probability systematic sampling according to proportional to EAs measure of size from January 18, 2016, to June 27, 2016. National representatives of 18,008 households were selected based on a nationally representative sample and 16,583 eligible women were included. In this study, 443 EAs from rural Ethiopia were used. All women were aged 15–49 years who had at least one child in the five years before the survey were eligible for participation. The children’s record data and their mother’s record data were merged to obtain a working dataset. The sample for this study would consist of 9254 under-five children from rural Ethiopia, from which only 7911 of them would be considered in this study. The detailed sampling procedure was presented in the measure EDHS report [10].

### Inclusion and exclusion criteria

Any child aged 0–59 months who lived with their family in rural Ethiopia and whose parents were willing to participate in the study met the study's inclusion criteria. Children whose mothers or caregivers refused to participate in the study, mothers/caregivers of children who were seriously ill during data collection and/or children on treatment for a confirmed severe respiratory illness, and children whose mothers provided incomplete information were all excluded.

### Data collection instrument and quality control

A structured and pre-tested questionnaire was utilized to collect data. Interviewers used tablet computers to record responses during the 2016 EDHS interviews. Bluetooth technology was installed on the tablets to enable distant electronic file transfer (transfer assignment sheets from team supervisors to interviewees and transfer completed copies from interviewers to supervisors) To ensure that the data was of good quality, data collectors were given training. Data quality control and fieldwork coordination were also taught to regional coordinators, field supervisors, and CAPI (computer-assisted personal interview) supervisors. The data collection was overseen by the research investigators on a daily basis. A protocol was written and given to data collectors that govern the survey's design, execution, and administration. Interviews were conducted in quiet, pleasant places at convenient times after data collectors were briefed. Furthermore, participants were encouraged to provide honest responses by describing the study's purpose and significance, as well as ensuring the confidentiality of the data they would provide. Completed surveys were checked for completeness and consistency on a daily basis.

### Measurement of variables

#### The response variable

In this study, the interest variable (y) was constructed as the occurrence of cough accompanied by short, rapid breathing in the 2 weeks preceding the survey. The result was measured as a binary variable, with a code of 1 if a child had ARI and 0 otherwise.

$$y_{i} = \left\{ {\begin{array}{*{20}c} {1, if\,the\, i^{th}\,child\, suffered\, from\, ARI\, in\, each\, rural\, region } \\ {0, otherwise} \\ \end{array} } \right.$$

#### Independent variables

**Fig. 1:**
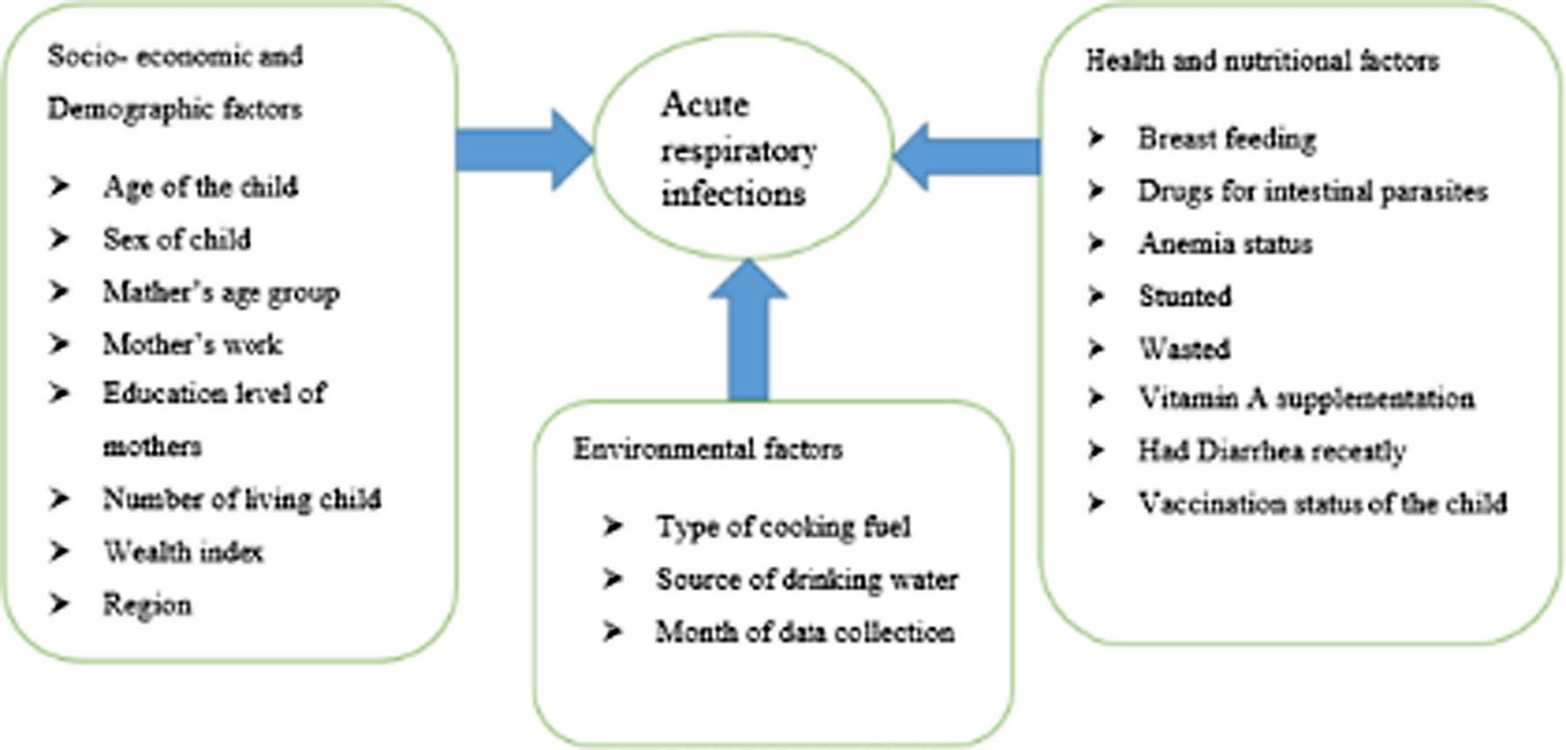
Conceptual framework to assess risk factors of acute respiratory infections among under-five children

All the explanatory variables were chosen based on existing literature and summarized in a conceptual framework (Fig. 1). Major explanatory variables included the nutritional index of the child, demographic and socio-economic factors, comorbidity disease, and environmental factors. Some of the variables were re-coded. The re-coding was done to make the results more understandable and to keep the sample size for the study at a manageable level. The nutritional status of children was determined by calculating z-scores for "height-for-age (stunting)" and "weight-for-height (wasting)" using WHO-recommended child physical growth indices [10, 27]. If the z-score for each nutritional status was two standard deviations below the median of the WHO reference population, children were declared stunted or wasted, according to WHO criteria [10, 27]. The age of the child was categorized as, less than 6 months, 6–11 months, 12–23 months, 24–35 months, 36–47 months, and 48–59 months; Sex of child; Mother’s age was defined as 15–29, 20–34 and 35 years and above; Household wealth was measured by a wealth index calculated using household assets data via principal component analysis as per the DHS guideline and categorized into five equal categories as poorest (1), poorer (2), middle (3), richer (4), and richest (5) and it is recorded as poor, middle and rich; Maternal education was divided into three categories: no education, primary and secondary education, and higher. Mothers' work was grouped into working and not working; Region; Number of a living child was expressed as 1–3 child, 4–6 child, and six and above [10, 25, 28].

Receipt of vitamin A in the last 6 months (Yes or No); Vaccination status was measured as ‘No’ for children who never had vaccine and ‘Yes’ for those who were vaccinated. Breast-feeding was assessed by asking mothers whether the child was exclusively breastfed for the first 6 months by classifying into never breastfeeding, ever breastfeeding, not currently, and still breastfeeding. Anemia status of the child (anemic/not anemic), the child had diarrhea (Yes or No) and children received no drugs for intestinal parasites in the last 6 months (Yes or No). Other categorizations of the explanatory variables were the source of drinking water, classified as “improved” or “not improved”. In the case of water piped into the home, piped to the yard/plot, public tap/standpipe, tube-well or borehole, protected well, rainwater, and bottled water, the source of drinking water is designated as "improved." If a drinking water source comprised of an unprotected well, unprotected spring, tanker truck/cart with drum, or surface water, it was classified as "not-improved." Whether the household uses improved cooking fuel (electricity, liquid petroleum gas, natural gas, biogas, kerosene, coal, lignite, charcoal) or unimproved (wood, animal dung, straw/shrubs/grass) fuel, and month of data collection was made from January 18 to June 27 [10, 29].

#### Statistical analysis

The analysis was done using SPSS version 20 software. Descriptive statistics and a multivariable logistic regression model were used to evaluate the data. To describe our study sample and to determine the prevalence of ARI in our study sample, descriptive statistics were used. The link between the response variable and the explanatory variables was investigated using a multivariable logistic regression model. Both bivariate and multivariable logistic regression analyses were performed. To choose candidate variables for multivariable logistic regression, bivariate logistic regression was used. In bivariate logistic regression, a p-value of less than 0.2 was used as a cut-off point. Before fitting the final model, the variance inflation factor (VIF) was employed to check for multi-collinearities amongst candidate variables. VIF values larger than 10 are typically assumed to indicate multi-collinearity, while values greater than 2.5 in weaker models, such as logistic regression, may be cause for concern [30]]. Based on the findings, all sets of variables in the current study have VIF values of less than 2.5, indicating that multi-collinearity is not an issue. Multivariable logistic regression model was employed to find the predictors of ARI in rural Ethiopian children under the age of five. To analyze the relationship between independent variables considered in the model and ARI, adjusted odds ratios (AOR) with 95% confidence intervals (CI) were generated. Finally, Hosmer and Lemeshow's test was employed to determine whether the fitted model was adequate. A p-value of less than 0.05 was judged to be statistically significant in this study's final model.

### Operational definitions

Acute respiratory infection was defined as a child will be considered as having experience of ARI if the mother reported that the child had a cough in the last two weeks before the survey date, accompanied by short rapid breathing. The symptoms are compatible with ARIs [10].

Co-morbidity was defined as the existence of one or more additional diseases co-occurring with primary disease, in this study comorbid diseases were drugs for intestinal parasites, anemia status, malnutrition, and diarrhea in under-five children.

## Results

The total number of children included in this study was 7911. From the sampled children, the two weeks’ prevalence of ARI among under-five children was about 7.8% in rural Ethiopia. Considering the ARI distribution by region, rural Oromia and rural Tigray account for the highest proportion of ARIs 12.8% and 12.7%**,** respectively, and lowest in rural Benishangul Gumuz 2.4% (Table 1).

**Table 1:** Socio-economic and demographic characteristics of respondents in rural Ethiopia from January 18 to June 27, 2016 (n = 7911)

Variables	Categories	Presence of ARI		Total	P-value
		No (%)	Yes (%)		
Sex	Male	3728 (92.2)	314 (7.8)	4042	0.816
	Female	3563 (92.1)	306 (7.9)	3869	
Age of the child	Less than 6 months	814 (92.0)	71 (8)	885	0.000
	6–11 months	694 (88.4)	91 (11.6)	785	
	12–23 months	1326 (89)	164 (11)	1490	
	24–35 months	1421 (91.8)	127 (8.2)	1548	
	36–47 months	1443 (93.2)	106 (6.8)	1549	
	48–59 months	1593 (96.3)	61 (3.7)	1654	
Maternal age	15–19	290 (91.8)	26 (8.2)	316	0.818
	20–34	5193 (92.1)	447 (7.9)	5640	
	35–49	1808 (92.5)	147 (7.5)	1955	
Wealth index	Poor	4670 (91.1)	458 (8.9)	5128	0.000
	Middle	1211 (92.9)	92 (7.1)	1303	
	Rich	1410 (95.3)	70 (4.7)	1480	
Mothers education level	No education	5262 (92.1)	451 (8.5)	5713	0.002
	Primary	1721 (91.5)	160 (7.9)	1881	
	Secondary and above	308 (97.2)	9 (2.8)	317	
Mothers currently working	Not working	5508 (92.4)	456 (7.6)	5964	0.19
	Working	1783 (91.6)	164 (8.4)	1947	
Number of living child	1–3 child	3264 (92.4)	267 (7.6)	3531	0.972
	4–6 child	2828 (91.8)	251 (8.2)	3079	
	6 and above	1199 (92.2)	102 (7.8)	1301	
Region	Rural Tigray	716 (87.3)	104 (12.7)	820	0.000
	Rural Afar	804 (92.9)	61 (7.1)	865	
	Rural Amhara	733 (90.3)	79 (9.7)	812	
	Rural Oromia	1217 (87.2)	179 (12.8)	1396	
	Rural Somali	1042 (95.2)	53 (4.8)	1095	
	Rural Benishangul Gumuz	725 (97.6)	18 (2.4)	743	
	Rural SNNPR	987 (92)	86 (8)	1073	
	Rural Gambela	473 (96.3)	18 (3.7)	491	
	Rural Harari	351 (96.7)	12 (3.3)	363	
	Rural Dire Dawa	243 (96)	10 (4)	253	
	Total	7261 (92.2)	620 (7.8)	7911	

**Fig. 2:**
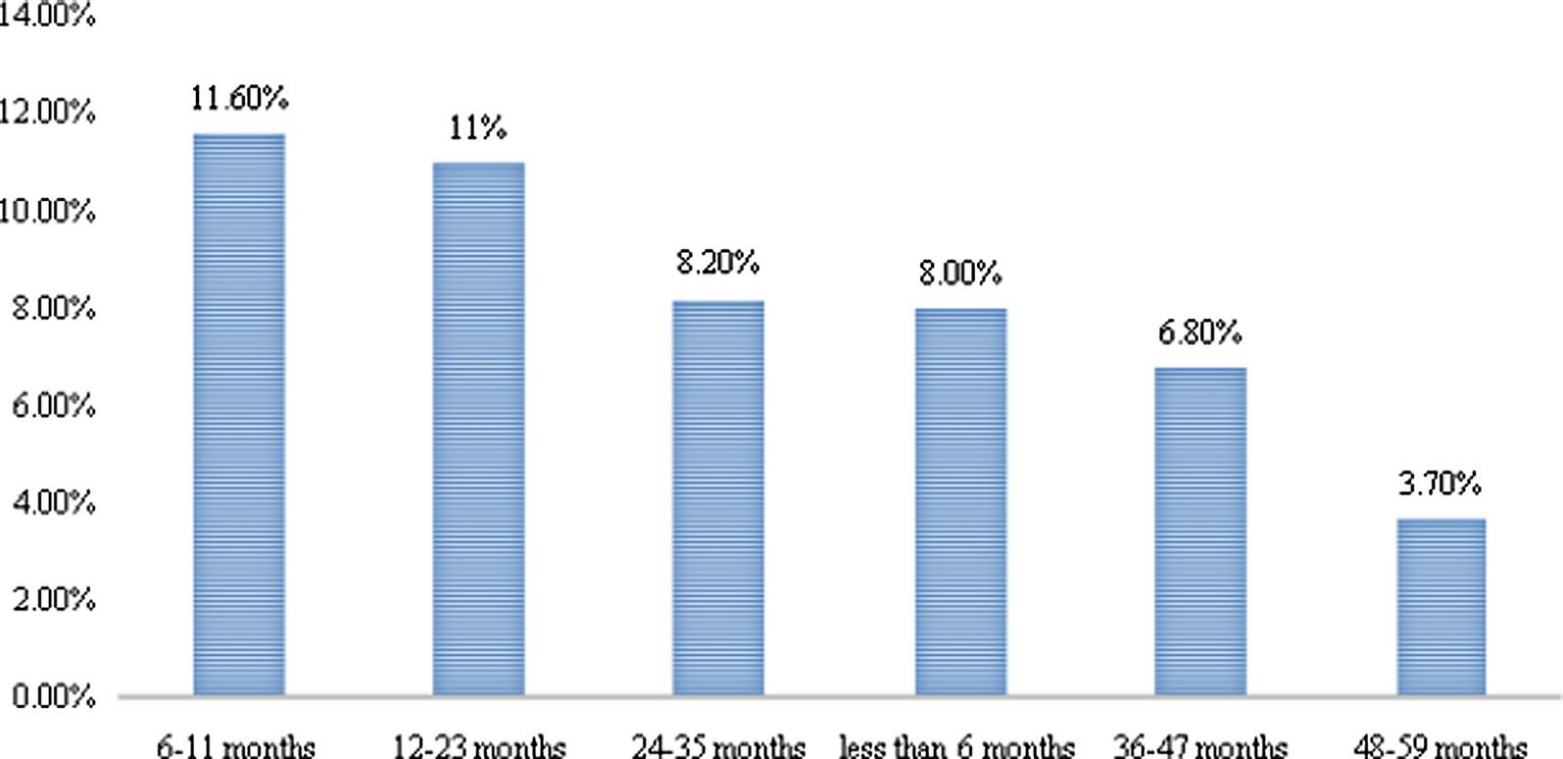
Distribution of ARIs according to the age of the child in rural Ethiopia

Out of 7911 under-five children the proportion of males and females were 4042 (51.1%) and 3869 (48.9%) with the prevalence of ARI 7.8% and 7.9% respectively. The prevalence of ARI was highest in the age group 6–11 months (11.6%), followed by 12–23 months (11%), and comparatively lower in the 48–59 months’ age group (3.7%) (Fig. 2).

Regarding maternal educational status, 5713(72.2%) had no education. In terms of the wealth index, 5128 (64.8%) of the participants were from poor families. Poor families have a larger percentage of under-five children with ARI (8.9%) (Table 1.

The child whose source of drinking water was unprotected/unimproved had the highest rate of ARI (9. 2%). In addition, the highest frequency of ARI was documented in January (12.5%), and the highest prevalence of ARI was recorded in households that utilized Unsafe/unclean cooking fuel (8.3%) (Table 2).

**Table 2:** Environmental characteristics of the respondents in rural Ethiopia from January 18 to June 27, 2016 (n = 7911)

Variables	Categories	Presence of ARI		Total	P-value
		No (%)	Yes (%)		
Type of cooking fuel	Unsafe/unclean	7247 (92.2)	616 (8.3)	7863	0.898
	Safe/clean	44 (91.7)	4 (7.8)	48	
Type of water source	Not improved	4714 (90.8)	476 (9.2)	5190	0.000
	Improved	2577 (94.7)	144 (5.3)	2721	
Month of data collection	January	496 (87.5)	71 (12.5)	567	0.000
	February	1647 (91.2)	158 (8.8)	1805	
	March	1780 (94.0)	113 (6.0)	1893	
	April	1658 (93.0)	124 (7.0)	1782	
	May	1532 (91.6)	140 (8.4)	1672	
	June	178 (92.7)	14 (7.3)	192	

Of the children, 899 (11.4%) had a history of diarrhea with the highest prevalence of ARI (21.9%). About 4662(58.9) were not vaccinated against any type of vaccine-preventable infectious with the highest prevalence of ARI (7.9%). Regarding nutritional status, 959 (12.12%) were wasted and 3129(39.5%) were stunted with the highest prevalence of ARI (9.9%) and (9.7%) respectively. The majority, 6655 (84.1%) of children received no drugs for intestinal parasites in the last 6 months, and 4707(59.5%) of them not received vitamin A recently with the highest proportion on the prevalence of ARI (9.5%) (Table 3).

**Table 3:** Nutritional and Comorbidity Characteristics of ARI among Under Five Children in rural Ethiopia from January 18 to June 27, 2016 (n = 7911)

Variables	Categories	Presence of ARI		Total	P-value
		No (%)	Yes (%)		
Duration of breastfeeding	Never breast feeding	272 (94.4)	16 (5.6)	288	0.000
	Ever breastfeeding, not currently	3978 (93.8)	261 (6.2)	4239	
	Still breast feeding	3041 (89.9)	343 (10.1)	3384	
Receive vitamin A in last 6 months	No	4259 (90.5)	448 (9.5)	4707	0.000
	Yes	3032 (94.6)	172 (5.4)	3204	
Had diarrhea recently	No	6589 (94)	423 (6)	7012	0.000
	Yes	702 (78.1)	197 (21.9)	899	
Acute malnutrition of child	No	6427 (92.4)	525 (7.6)	6952	0.011
	Yes	864 (90.1)	95 (9.9)	959	
Chronic malnutrition of a child	No	4465 (93.4)	317 (6.6)	4782	0.000
	Yes	2826 (90.3)	303 (9.7)	3129	
Drugs for intestinal parasites	No	6128 (92.1)	527 (7.9)	6655	0.534
	Yes	1163 (92.6)	93 (7.4)	1256	
Anemia status of the child	Not anemic	4575 (93.4)	323 (6.6)	4898	0.000
	Anemic	2716 (90.1)	297 (9.9)	3013	
Vaccination status	No	4293 (92.1)	369 (7.9)	4662	0.758
	Yes	2998 (92.3)	251 (7.7)	3249	

### Factors associated with acute respiratory infection among under-five children

In multivariable logistic regression analysis, age of the child, wealth index of household, mother’s education level, had received vitamin A recently, had diarrhea, stunted, source of drinking water, maternal occupation, the month of data collection, anemia status, and the region were significantly related to the acute respiratory infections of under-five children.

The impact of many factors on childhood ARI status was assessed using a multivariable logistic regression model, and the significant findings are summarized in the following section. Based on a multivariable logistic regression analysis, the odds of ARI decreased as the age of the child increased. The odds of incidence of ARI among under-five children in the age group of less than 6 months,6–11 month, 12–23 month,24–35 month, 36–47 month, were (AOR = 1.936, 95% CI: 1.243–3.014),(AOR = 2.783,95%CI:1.806–4.288),(AOR = 2.47095%CI:1.697–3.597), (AOR = 1.987, 95% CI:1.420–2.779) and (AOR = 1.729, 95% CI:1.238–2.416) times more likely experiencing ARI than those children in the age group 48–59 months respectively (Table 4).

**Table 4:** Associations between ARI and predictor variables among children under five in rural Ethiopia from January 18 to June 27, 2016 (n = 7911)

Variables	Categories	Presence of ARI		COR(95% CI)	AOR (95% C.I)
		No	Yes		
Age of the child	48–59 months (ref)	1593	61	1.0	1.0
	Less than 6 months	814	71	2.278 (1.601–3.240)	1.936 (1.243–3.014)*
	6–11 months	694	91	3.424 (2.446–4.793)	2.783 (1.806–4.288)*
	12–23 months	1326	164	3.230 (2.386–4.372)	2.470 (1.697–3.597)*
	24–35 months	1421	127	2.334 (1.706–3.194)	1.987 (1.420–2.779)*
	36–47 months	1443	106	1.918 (1.389–2.650)	1.729 (1.238–2.416)*
Wealth index	Rich (ref)	1410	70	1.0	1.0
	Poor	4670	458	1.975(1.526–2.558)	2.170(1.631–2.887)*
	Middle	1211	92	1.530(1.111–2.108)	1.388(0.992–1.943)
Duration of breastfeeding	Still breastfeeding (ref)	3041	343	1.0	1.0
	Never breast feeding	272	16	0.522(0.311–0.874)	0.884(0.507–1.544)
	Ever breastfeeding, not currently	3978	261	0.582 (0.492–0.688)	0.942 (0.724–1.226)
Mothers educational level	Secondary and above (ref)	308	9	1.0	1.0
	No education	5262	451	2.933 (1.501–5.731)	2.050 (1.017–4.133)*
	Primary	1721	160	3.182 (1.608–6.295)	2.387 (1.176–4.845)*
Receive vitamin A in last 6 months	Yes (ref)	3032	172	1.0	1.0
	No	4259	448	1.854 (1.546–2.224)	1.926 (1.578–2.351)*
Had diarrhea recently	Yes (ref)	702	197	1.0	1.0
	No	6589	423	0.229 (0.190–0.276)	0.257 (0.210–0.314)*
Mothers currently working	Working (ref)	1783	164	1.0	1.0
	Not working	5508	456	0.9 (0.747–1.084)	0.773 (0.630–0.948)*
Wasted	Yes (ref)	864	95	1.0	1.0
	No	6427	525	0.743 (0.590–0.935)	0.788 (0.614–1.011)
Stunted	Yes (ref)	2826	303	1.0	1.0
	No	4465	317	0.662 (0.562–0.780)	0.663 (0.552–0.796)*
Type of water source	Improved (ref)	2577	144	1.0	1.0
	Not improved	4714	476	1.807 (1.491–2.191	1.715 (1.395–2.109)*
Region	Rural Dire Dawa (ref)	243	10	1.0	1.0
	Rural Tigray	716	104	3.530 (1.815–6.863)	3.628 (1.817–7.244)*
	Rural Afar	804	61	1.844 (0.930–3.653)	1.446 (0.711–2.941)
	Rural Amhara	733	79	2.619 (1.335–5.137)	2.410 (1.192–4.872)*
	Rural Oromia	1217	179	3.574 (1.863–6.857)	3.516 (1.784–6.928)*
	Rural Somali	1042	53	1.236 (0.620–2.464)	1.132 (0.553–2.317)
	Rural Benishangul gumuz	725	18	0.603 (0.275–1.325)	0.545 (0.241–1.230)
	Rural SNNPR	987	86	2.117 (1.084–4.137)	2.377 (1.181–4.784)*
	Rural Gambela	473	18	0.925 (0.420–2.034)	0.832 (0.367–1.886)
	Rural Harari	351	12	0.831 (0.353–1.953)	0.805 (0.333–1.944)
Month of data collection	June(ref)	178	14	1.0	1.0
	January	496	71	1.820 (1.001–3.310)	2.125 (1.127–4.010)*
	February	1647	158	1.220 (0.691–2.152)	1.535 (0.841–2.801)
	March	1780	113	0.807 (0.454–1.436)	0.989 (0.538–1.819)
	April	1658	124	0.951 (0.536–1.688)	1.231 (0.670–2.260)
	May	1532	140	1.162 (0.656–2.056)	1.319 (0.720–2.414)
Anemia status	Anemia (ref)	2716	297	1.0	1.0
	Not anemic	4575	323	0.646 (0.548–0.761)	0.636 (0.532–0.761*

*Significant at $p < 0.05$. Ref = reference category, Hosmer and Lemeshow's goodness of model test was found to be chi-square of 19.614 with a p-value of 0.12 which implies the goodness of the model to predict the outcome

The odds of under-five children developing ARI among mothers who had no education to have ARI was 2.050 (AOR 2.050; 95% CI 1.017–4.133) times more likely affected by ARIs as compared with children whose mothers’ had secondary and above educational level and children whose mothers had primary education was 2.387 times more likely compared with children whose mothers had secondary and above education level (AOR 2.387; 95% CI 1.176–4.845). Moreover, children within the poorest quintile were 2.170 times more likely to develop cases (AOR = 2.170, 95% CI: 1.631–2.887), compared with children from the richest households.

The odds of ARI among not stunted children were 33.7% less likely to develop ARI compared to stunted children (AOR 0.663; 95% CI 0.552–0.796). Maternal occupation also has a statistically significant association with ARI; accordingly, compared with children of employed mothers, children whose mothers were not worked had 22.7% reduced odds of having ARI (AOR = 0.773; 95% CI 0.630–0.948).

According to this study, Vitamin A consumption of children in the last six months was found that it had a significant effect on the ARIs. The odds of a child who had not received vitamin A recently was 92.6% (AOR = 1.926, 95% CI 1.578–2.351) more likely to suffer from ARI compared to a child who received vitamin A recently. Indeed, the odds of Under-five children who had no Diarrhea recently were 74.3% (AOR = 0.257, CI: 0.210–0.314) times less likely to experience ARI compared with a child who had Diarrhea recently. Anemia has been stated as one of the risk factors of ARI. When compared to a kid who had anemia, the child who did not have anemia was 36.4% (AOR = 0.636 95 percent CI: 0.532–0.761) less likely to develop ARI and the frequency of ARI was strongly linked to the season, the prevalence of ARI in January was 2.125 times (AOR = 2.125 95% CI:1.127–4.010) higher than in the reference group.

The source of drinking water also has a significant effect on ARI. The odds of children using an unprotected source of drinking water were 71.5% (AOR = 1.715, 95% CI: 1.395–2.109) times more likely to have ARI as compared with a child using a protected source of drinking water. Province was also significantly related to ARI symptoms (P < 0.05). The odds of having ARI symptoms for children in rural Afar, rural Somali, rural Benishangul Gumuz, rural Gambela, and rural Harari were not significantly different as compared to rural Dire Dawa. Children in rural Tigray regional state (AOR: 3.628, 95% CI: 1.817–7.244), Children who lived in rural Amhara (AOR: 2.410, 95% CI: 1.192–4.872), rural Oromia (AOR: 3.516, 95% CI: 1.784–6.928), and rural South Nation Nationalities Peoples regional states (AOR: 2.377, 95% CI: 1.181–4.784) were significantly higher risk of ARI symptoms as compared to rural Dire Dawa.

## Discussions

The current study aimed at evaluating the prevalence of, and risk factors associated with, AR in rural Ethiopia based on the Ethiopian Demographic and Health Survey data. In the study overall prevalence of ARI was found to be 7.8%. Our findings are comparable to those of a study conducted by [31] in Zambia, where the prevalence of ARI was reported to be 8% and 6.9% in Iraq by [32]. In contrast to our findings, a study conducted in New Delhi slums estimated the overall prevalence of ARI among children under the age of five to be around 4.5% for one month [33]. ARI affected 21.3% of Bangladeshi children under the age of five in the two weeks before the survey [17]. Differences in study populations, study sites, age categories investigated, the method used to assess the outcome variable, comorbidities, and variations in the study period and season could all contribute to the variation in ARI proportions.

The study showed that there is an enormous disparity in the distribution of ARIs in children across the different regional states of rural Ethiopia. According to the descriptive data analysis, the highest prevalence of ARI occurred in rural Oromia and rural Tigray at 12.8% and 12.7% respectively and the lowest, 2.4%, in rural Benishangul Gumuz region. It is in line with [34, 35]. There could be numerous reasons for the regional variations in illness distributions; high-risk areas for ARI were found in the northern and central parts of the country, particularly rural Tigray and rural Oromia. These were highland areas, implying a high prevalence of ARI among children under the age of five. Children are indoors for longer periods when the weather is cold, putting them in close contact with a variety of bacteria, fungi, and viruses. When the temperature drops, flu, viruses, and bacteria are more likely to remain stable in the air, forming respiratory droplets and causing damage to the airways, allowing bacteria to cause infections, most commonly ARI in the lungs. Another reason could be that many of the children in these areas were under the age of one year, and the majority of households in these areas relied on charcoal and cow dung as a source of fuel.

In this study, age of the child, wealth index of household, maternal education, received vitamin A supplement, had diarrhea recently, stunting, maternal occupation, source of drinking water, anemia status, the month of data collection, and region of the residence were found to be significantly associated with ARI symptoms. Children aged 48–59 months have a lower risk of developing ARI symptoms. This finding is consistent with studies undertaken in Ahmadabad city and other low- and middle-income nations [36, 37]. The prevalence of ARI is particularly common among children aged 6–36 months, according to studies conducted in the People's Republic of China and South India [38, 39]. Compared to a child more than 48 months, the chances of the child at the earlier age experiencing acute respiratory infection symptoms are higher. It is obvious that as a child grows older, he or she will have greater resistance to diseases such as cough and diarrhea [14, 25]. Because children's immunity develops as they grow older, they are more prepared to battle infection.

Children from the poorest families are more likely to contract ARI than those from wealthier households. These findings are supported by the study done in Bangladesh, residence in poor households conferred 1.3 times (95% CI 1.09–1.55) higher odds of ARI in young children [17]. Under-five children those whose family were from the low socio-economic class was also significantly associated with ARI [40–43]. This is due to wealthier households tend to afford better nourishment and health care for their children. Wealthier families can also minimize their children’s exposure to risk factors like unsanitary environments and contaminated water [44]. These findings are supported by other studies which found that higher poverty levels increase the risk of ARI and diarrhea [45, 46]

Children whose mothers have no or only a primary education have a much higher risk of ARI than children whose mothers have a secondary or higher degree of education. This statement is consistent with earlier findings [14, 47]. This could be because education has improved mothers' ability to apply basic health knowledge and has facilitated their ability to manipulate their environment, including health care facilities, work more effectively with health professionals, follow treatment recommendations, and keep their environment clean. Furthermore, educated mothers have more power over their children's health decisions.

Children with stunted were shown to have a greater prevalence of ARI. This finding is also in line with studies conducted in India, specifically in Solapur and Gujarat, Ethiopia, and other developing countries [22, 36, 39, 43, 48–50]. This finding indicates that malnourished children have inadequate immunity and are susceptible to a variety of diseases, including ARI.

A kids who had not recently received vitamin A was 92.6% more likely to develop ARI than a child who had recently received vitamin A. Promoting Vitamin A supplementation for all rural children could improve their health. Several studies backed up our conclusions [17].

In this study, under-five children from rural areas with a history of diarrhea were more affected by ARI. This research backed up a study conducted in northeast Ethiopia's Oromia zone, which found that children with a history of diarrhea were three times more likely to get ARI than their peers [51]. Similar studies have been carried out in southwest Ethiopia and Ghana [52, 53]. In addition, a study done in Bangladeshi found that children with a history of diarrhea were more likely to develop ARI [54]. The reason is children who have a concomitant illness like diarrhea may have a lowered immunity, making them more susceptible to a disease like ARI. The reason for this is that children with a comorbid ailment, such as diarrhea, anemia may have decreased immunity, making them more vulnerable to diseases like ARI.

In rural Ethiopia, the source of drinking water is another key environmental element that influences the ARI of children under the age of five. This study found that children who drink water from an unprotected/unimproved source are more likely to have ARI symptoms than children who drink water from a protected/improved source. This result was supported by [21, 55]. This was due to the fact that contaminated water was thought to be the leading cause of acute respiratory infection.

Mother’s occupation was found to be a significant factor of ARIs. Compared to children of mothers currently working those children whose mothers have no work in any sectors have a significantly lower risk of ARI. This means mothers to stays at home may keep their child in a good and clear environment. This result was supported by [14, 34]. The other explanation might be that, mothers who had work could be exposed to certain chemicals, pollutants, or toxic fumes within the working environment, thereby transmitting the infection to their children may be increased. Furthermore, because the majority of mothers in developing countries like Ethiopia work in the informal sector and lower-status occupations, the amount of income for these mothers is low and would be a significant effect on ARIs of under five children.

The odds of the child suffering from ARI were higher in January. Because this study was carried out during the peak of the dry season which is characterized by dry and dusty harmattan winds. It is consistent with the previous study done by [56–58]. Anemia or low hemoglobin status has also been stated as one of the risk factors of ARI. This finding was consistent with the studies conducted by [59]. Similar significance was also shown in other studies like [60, 61]. Because most healthy children can fight infection with their natural defenses, children with impaired immune systems are more susceptible to infection. Anemia amplifies this effect by reducing the body's natural defenses. So, when the current study was compared to previous similar studies, there was a strong link between anemia and ARI. As a result, preventing anemia and detecting anemia early can help to lower the incidence of acute respiratory tract infection.

Finally, ARI among under-five children was a significant association with regions. It revealed that the probability of children living in rural Tigray, rural Amhara, rural Oromia, and rural SNNPR regions have higher ARI symptoms before two weeks preceding the survey than children who living in rural Dire Dawa regions. It is consistent with a previous study done by [34]. While, the odds of children living in rural Somali, rural Benishangul-Gumuz, rural Gambela, and rural Afar, being have ARI symptoms before two weeks are significantly not different from those living in rural Dire Dawa.

### Strengths and weaknesses of the study

A strength of this study was that it assessed many important determinant factors including socioeconomic status, maternal and child nutrition, environmental, health, and other factors contributing to ARI in a cross-sectional study design. This study's findings should help policymakers better grasp this rapidly evolving health hazard and adopt appropriate legislative steps. Aside from our significant contributions, our research contains a number of flaws. First, the symptoms of ARIs were reported by mothers and were not the result of clinical examinations. Because the variables were self-reported, they are prone to reporting bias. Second, no data on household hygiene practices, which is a critical predictor of infectious diseases in people of all ages, was available. However, this gap is expected to be overcome, to a great extent, by the inclusion of sanitation variables. Finally, because the data was secondary, no causal inferences regarding the correlations could be drawn. Therefore, the reader of this article should take into account the above barriers.

## Conclusions

In this study from a rural setting in Ethiopia, significant factors for ARI in under-five children were child age, maternal/caregiver occupation, economic status of the family, received vitamin A recently, had diarrhea recently, anemia status, the month of data collection, region, mother’s education level, source of drinking water and nutritional status of the child. The occurrence of ARI could be reduced by implementing interventions to improve economic status, nutrition status, mother’s educational level and by increasing community awareness regarding protected water, diarrhea, anemia, morbidity, and vitamin A supplementation for the prevention of ARI amongst under-five children.

## Data Availability

The datasets generated and analyzed during the present study are available from the corresponding author on reasonable request.

## Abbreviations

ALRI: Acute Lower Respiratory Infections
AOR: Adjusted Odds Ratio
ARI: Acute Respiratory Infections
AURI: Acute Upper Respiratory Infections
CI: Confidence Interval
CSA: Central Statistical Agency
EA: Enumeration Areas
EDHS: Ethiopia Demographic Health Survey
FMoH: Federal Ministry of Health
SNNPR: South Nations Nationalities Peoples Region
WHO: World Health Organizations
